# Phytohormone targeting in plant tissues

**DOI:** 10.1186/1753-6561-5-S7-P75

**Published:** 2011-09-13

**Authors:** Miroslav Strnad, Ondrej Novak, Jakub Rolcik, Danuse Tarkowska, Jiri Gruz, Karel Dolezal

**Affiliations:** 1 Laboratory of Growth Regulators, Faculty of Science, Palacky University & Institute of Experimental Botany AS CR, Slechtitelu 11, Olomouc, CZ-783 71, Czech Republic

## Background

The identification and quantification of plant hormones in plant tissues are necessary for physiological studies of their metabolism and mode of action. The major problem associated with plant hormone analysis is that the amount of phytohormones present endogenously in plant tissues is very low, usually in the range of fmol to pmol/g fresh weight.

## Methods

Homogenization and extraction with organic solvents was done in one microcentrifuge tube and accelerated by crushing the plant material in a vibration mill. The extracts from minute amounts of fresh plant material were immediately purified using a solid-phase extraction (C18, C8, Oasis™ HLB cartridges)in combination with ion-exchange and/or immunoaffinity purification. A fast chromatography technique, the ultra performance liquid chromatography (Acquity^TM^ UPLC,Waters) was coupled to triple quadrupole mass spectrometer (Xevo^TM^ TQ MS, Waters) equipped with an electrospray interface (ESI) and the unique performance of collision cell – ScanWave^TM^. The mass spectrometric conditions were optimized for each analyte and quantification was obtained by multiple reaction monitoring (MRM) of precursors and the appropriate product ions.

## Results and discussion

We found that a combination of different sorbents, reverse phases and ion-exchange phases, was the best tool in the one-step purification, giving a total extraction recovery ranging between 50-80% for all studied biologically active compounds. In MRM mode, the detection limit for most of phytohormones (cytokinins [1,2], auxins [3], abscisic acid [4], gibberellins, brassinosteroids) as well as phenolic acids [5] and mammalian steroids [6] was close to 1 fmol and achieved linear range was at least five orders of magnitude. Use of our procedures can allow the quantification of plant hormones and their derivatives (in total 145 compounds) in very limited amounts of material, ca. 100 mg FW. The methods provide substantial improvements in terms of robustness, sensitivity, selectivity, convenience, through-put and cost-effectiveness over previous methods published.

## Conclusions

The application of new analytical approaches based on UPLC separation makes possible a new direction in plant hormone research. We believe that UPLC-ESI(+)MS/MS technology can be used for fast and sensitive quantitative analysis showing reproducibility in the plant hormones profiling in different tree extracts.
